# Use of machine learning models to predict mechanical ventilation, ECMO, and mortality in COVID-19

**DOI:** 10.3389/frai.2025.1661637

**Published:** 2026-01-06

**Authors:** Nina Moorman, Erin Hedlund-Botti, Grace Gombolay, Matthew C. Gombolay

**Affiliations:** 1 Georgia Institute of Technology, Atlanta, GA, United States; 2 Division of Neurology, Children’s Healthcare of Atlanta, Atlanta, GA, United States; 3 Division of Pediatric Neurology, Department of Pediatrics, Emory University School of Medicine, Atlanta, GA, United States

**Keywords:** machine learning, artificial intelligence, predictors, COVID-19, coronavirus-19

## Abstract

**Introduction:**

Patients with severe COVID-19 may require MV or ECMO. Predicting who will require interventions and the duration of those interventions are challenging due to the diverse responses among patients and the dynamic nature of the disease. As such, there is a need for better prediction of the duration and outcomes of MV use in patients, to improve patient care and aid with MV and ECMO allocation. Here we develop and examine the performance of ML models to predict MV duration, ECMO, and mortality for patients with COVID-19.

**Methods:**

In this retrospective prognostic study, hierarchical machine-learning models were developed to predict MV duration and outcome prediction from demographic data and time-series data consisting of vital signs and laboratory results. We train our models on 10,378 patients with positive severe acute respiratory syndrome-related coronavirus (SARS-CoV-2) virus testing from Emory’s COVID CRADLE Dataset who sought treatment at Emory University Hospital between February 28, 2020, to January 24, 2022. Analysis was conducted between January 10, 2022, and April 5, 2024. The main outcomes and measures were the AUROC, AUPRC and the F-score for MV duration, need for ECMO, and mortality prediction.

**Results:**

Data from 10,378 patients with COVID-19 (median [IQR] age, 60 [48–72] years; 5,281 [50.89%] women) were included. Overall MV class distributions for 0 days, 1–4 days, 5–9 days, 10–14 days, 15–19 days, 20–24 days, 25–29 days, and ≥30 days of MV were 8,141 (78.44%), 812 (7.82%), 325 (3.13%), 241 (2.32%), 153 (1.47%), 97 (0.93%), 87 (0.84%), and 522 (5.03%), respectively. Overall ECMO use and mortality rates were 15 (0.14%) and 1,114 (10.73%), respectively. On MV duration, ECMO use, and mortality outcomes, the highest-performing model reached weighted average AUROC scores of 0.873, 0.902, and 0.774, and the highest-performing model reached weighted average AUPRC scores of 0.790, 0.999, and 0.893.

**Conclusions and relevance:**

Hierarchical ML models trained on vital signs, laboratory results, and demographic data show promise for the prediction of MV duration, ECMO use, and mortality in COVID-19 patients.

## 1 Introduction

Coronavirus disease 2019 (termed COVID-19) is caused by the severe acute respiratory syndrome-related coronavirus (SARS-CoV-2) virus (About COVID-19, 2024). There have been more than 775 million cases and 7 million deaths confirmed due to COVID-19 as of May 20, 2024 (Cumulative confirmed COVID-19 cases and deaths, World, n.d.). Patients with severe COVID-19 may require mechanical ventilation (MV) or extracorporeal membrane oxygenation (ECMO) and are at risk for mortality (Shaefi et al., 2021). While MV may be lifesaving (Cronin et al., 2022; Bellani et al., 2021), MV can result in injury and other complications (Butler et al., 2023; Esteban et al., 2013; Loss et al., 2015). Thus, predictors of outcomes in COVID-19 are critical for the management of patients with COVID-19 and can aid in allocated limited resources (Santini et al., 2022).

Researchers have sought to develop data-driven mechanisms to predict outcomes in COVID-19, including developing heuristic scoring systems heuristic (Shah et al., 2023; Supady et al., 2021; Garcia-Gordillo et al., 2021; Kafan et al., 2021). More sophisticated computational models have been developed to predict the need for (Shashikumar et al., 2021) and duration of MV (Kobara et al., 2023; Ryan et al., 2020; Taleb et al., 2021), mortality (Ryan et al., 2020; Ohshimo et al., 2022) and intensive care unit (ICU) duration (Taleb et al., 2021). Machine learning (ML) algorithms have also predicted adverse outcomes in COVID-19 (Yu et al., 2021; Bendavid et al., 2022; Douville et al., 2021; Lorenzoni et al., 2021; George et al., 2021; Kim et al., 2021). However, none of these approaches have holistically combined the prediction of these outcome metrics to systematically understand the course of COVID-19 patients. Instead, prior work has modeled MV usage and duration, ECMO usage, or mortality as distinct phenomena (Rodriguez et al., 2021; He et al., 2022).

In this work, we develop a long short-term memory artificial recurrent neural network approach (RNN), which naturally encodes time-series information, that integrates patient demographics and time-series vitals and laboratory values for jointly predicting MV and ECMO use, MV duration, and mortality. Our unique approach is hierarchical in that it makes sequential predictions that are subsequently used for more predictions. This hierarchy provides a helpful inductive bias for model training, helping the model to learn to think step-by-step. On a novel dataset of 10,378 COVID-19 patients, we find that our RNN-based approach outperforms standard ML baselines. Unlike prior work (Rodriguez et al., 2021; He et al., 2022), our approach encodes time-series data in a flexible graphical model, which can improve model performance and enable real-time predictions with streaming data. Further, we include ECMO as a distinct outcome from MV unlike some prior work (He et al., 2022; Zayat et al., 2021; Tabatabai et al., 2021; Dreier et al., 2021) as it is associated with mortality (Henry and Lippi, 2020).

Further, we inspect the reasoning of our approach through feature permutation importance (PI) and SHapley Additive exPlanations (SHAP) to gain clinical insights. We propose that our ML modeling could be helpful for clinical decision- making for individual patients in deciding the need for and the length of MV. Moreover, these models could help with resource utilization, by predicting the number of patients in a hospital who will require MV and the duration of MV, along with the need for ECMO, which could help the staff prepare to have the necessary equipment to manage patients with COVID-19.

## 2 Methods

### 2.1 Source of data

This study was conducted on the COVID dataset composed of electronic health records of protected health information provided by Emory University, as part of the CRADLE (Emory Clinical Research Analytics Data Lake Environment) Project. We followed the Transparent Reporting of a multivariable prediction model for Individual Prognosis or Diagnosis (TRIPOD) reporting guidelines.

### 2.2 Study cohort

Our single-center study cohort was selected from the 41,319 patients at Emory Healthcare (EHC) diagnosed with COVID-19 between February 1, 2020 and January 24, 2022. Additionally, patients must meet at least one of the following eligibility criteria:

1. A positive/detected lab result verified on/after February 1, 2020 for one of the following (1) SARS-CoV-2 PCR completed at either Emory University Hospital (Emory), ARUP Laboratories |National Reference Laboratory (ARUP), or Quest Diagnostics (Quest); (2) SARS-Cov-2 RNA completed at EHC.
2. A positive SARS-CoV-2 test conducted by an outside lab and documented on the COVID-19 Non-EHC Labs power form with a service date on or after February 1, 2020.
3. An International Classification of Diseases, Tenth Revision (ICD-10) code of U07.1 captured as the primary or secondary billing diagnosis from Medical Records Coding or the Charms 2000 PowerAbstract system (Vendor: Meta Health Technology Inc.) for encounters not considered long-term care or hospice discharged on or after April 1, 2020 from Emory- related hospitals: Emory Johns Creek (EJCH), EUH, Wesley Woods, EUH Midtown (EUHM), or Emory Saint Joseph’s Hospital (ESJH).

We restricted this cohort to those whose hospital stay was at least 3 days and had at least one documented measurement for all feature types in Section, yielding 10,378 patients.

### 2.3 Data selection and preparation

#### 2.3.1 Features

We consider two types of features: dynamic and static. Dynamic features are vitals values and laboratory results measured over the first 3 days of the hospital stay. These features include Oxygen (O_2_) Saturation, Temperature (C), the fraction of inspired oxygen (FiO_2_), oxygen flow rate, heart rate (HR), sitting systolic BP, and sitting diastolic BP. Static features consist of demographics (i.e., race, ethnicity, age, and gender), BMI, and weight (see Supplementary material). These features are associated with COVID-19 outcomes (Bonaventura et al., 2022; Kimhi et al., 2020; Dhanani and Franz, 2022).

#### 2.3.2 Labels

Our models predict a probability distribution over the following: (1) MV duration (days) in ranges: 0 (i.e., no MV), 1–4, 5–9, 10–14, 15–19, 20–24, 25–29, and ≥30 days; (2) ECMO use; and (3) in-hospital mortality.

#### 2.3.3 Dataset preparation

For patient confidentiality, our dataset did not include admission/discharge times. We heuristically distinguished visits based on these criteria: (1) There must be at least 3 days (not necessarily consecutive) of feature information collected for that individual, to constitute a hospital visit; (2) No more than 3 days may elapse between measurements for that individual before the measurement is assigned to a new hospital visit; and (3) Each individual’s hospital stay must contain at least one measurement of all feature types to be included. After filtering, the number of hospital visit data points was reduced from 33,552 to 23,174.

We choose only to leverage the first three consecutive days’ worth of feature data in keeping with prior work (Hu et al., 2020). Further, we limited the number of measurements of each dynamic feature type to the first 100 measurements. If a patient had fewer than 100 dynamic measurements, we padded the feature array with zeros.

### 2.4 Model development

**Figure 1:**
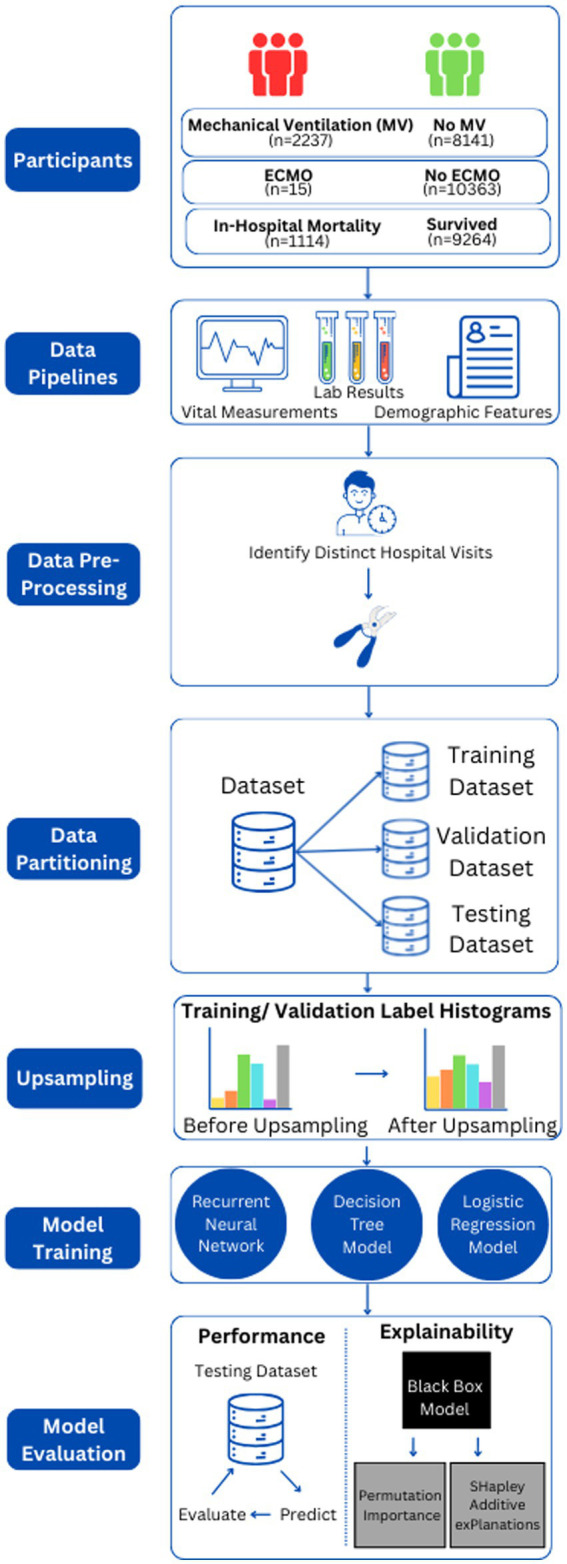
This figure depicts an overview of the pipeline, from data collection and preparation to model evaluation.

We randomly separated our dataset into a training (60%), validation (20%), and hold-out testing (20%) datasets. The training dataset was used to train our ML models, and the testing dataset was used for hyper-parameter selection (see Supplementary material). We report performance on the hold-out dataset. Figure 1 depicts our data analysis pipeline.

We note that it is possible that the same patient was admitted to the hospital more than once. Because we defined each “visit” using our own criteria and treated visits as independent data points, patient identifiers were not retained in the final dataset. As a result, a patient could have data from different visits appear in both the training and test sets. We recognize this as a limitation, but also note that it reflects real-world clinical practice, where models are often applied across multiple admissions for the same patient.

We provide a quantitative estimate of the impact of this overlap. To do so, we generate a new dataset from our database with the additional feature of participant id and assess variability in participant overlap between the training and test datasets by repeating the data-splitting procedure 1,000 times. We note that the data was de-identified, and no patient identifiers were accessible after pre-processing. Across these splits, we find that an average of 4.19 ± 0.39% of data in the test dataset (87.07 ± 8.09 / 2076) originated from patients who also appear in the training dataset.

#### 2.3.1 Recurrent neural network

**Figure 2:**
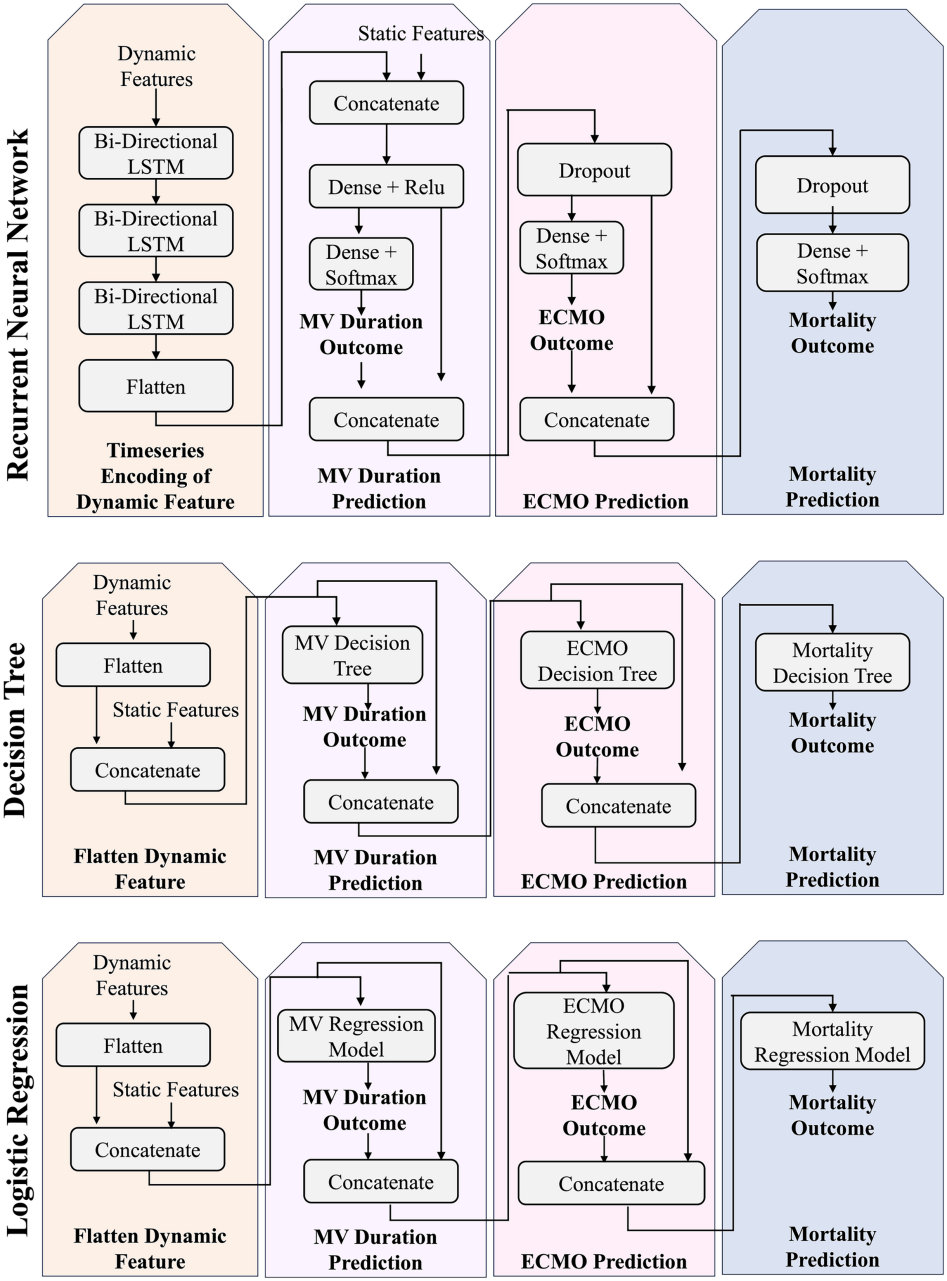
This figure depicts the hierarchical RNN architecture as well as the model-chained decision tree and logistic regression architectures.

LSTM neural networks are a type of recurrent neural network, which have been used for COVID-19 modeling (Rasmy et al., 2022; Kumar et al., 2021; Sun et al., 2021; Villegas et al., 2023) due to their ability to capture long-term dependencies and temporal patterns (Hochreiter and Schmidhuber, 1997). We employ TensorFlow’s Keras API to create our custom RNN (Abadi et al., 2015). As depicted in Figure 2, our hierarchical RNN first leverages bi-directional LSTM layers to capture the temporal dependencies in our sequential dynamic features. This is concatenated with the static feature data and leveraged to predict MV duration. Next, using both the predicted MV duration and the hidden activations (i.e., the output of a dense layer with ReLU activation), we predict the ECMO outcome. Finally, using the predicted ECMO outcome and the next set of hidden activations, we predict mortality. This architecture predicts these three outcomes and combines their cross-entropy losses into an overall loss to train the model.

#### 2.4.2 Decision tree

We train a set of decision tree (DT) classifiers, as per prior work (Ryan et al., 2020; Yu et al., 2021; Bendavid et al., 2022; Douville et al., 2021; Elhazmi et al., 2022), using sklearn (Pedregosa et al., 2011; Buitinck et al., 2013). As in Figure 2, the architecture includes three DT classifiers, each trained separately. As with our RNN approach, each prediction of the DT is passed as input to the next DT module along with the dynamic and static features. Hyperparameter optimization is performed, as described in the Supplementary material.

#### 2.4.3 Logistic regression

As depicted in Figure 2, we leverage logistic regression (LR) with elastic net regularization (Takada et al., 2022) using sklearn (Pedregosa et al., 2011; Buitinck et al., 2013) and perform a grid search over the C and L1 ratio hyperparameters (See Supplementary material).

#### 2.4.4 Metrics

We employ four metrics based upon prior work (Hicks et al., 2022; Saito and Rehmsmeier, 2015): (1) the area under the receiver operating characteristic (AUROC), area under the precision recall curve (AUPRC) (2) precision, (3) recall, and (4) F-score. Due to our imbalanced dataset, we do not report accuracy as it yields misleadingly high performance.

#### 2.4.5 Statistical analysis

We assessed the performance of three models in this study (i.e., the RNN, DT, and LR) on a holdout dataset. As our dataset has heavily unbalanced classes, we report class-specific metrics, weighted averages, and macro-averages (Zhang and Yang, 2003).

## 3 Results

### 3.1 Demographic and clinical characteristics

Table 1 show the demographics and outcomes of our cohort. Among 10,378 patients (median [IQR] age, 60 [48–72] years; 5,281 (50.89%) female and 5,097 (49.11%) male) included in our analysis, 0.14% experienced ECMO, 10.73% died in hospital. 78.44, 7.82, 3.13, 2.32, 1.47, 0.93, 0.84, and 5.03% experienced 0 days, 1–4 days, 5–9 days, 10–14 days, 15–19 days, 20–24 days, 25–29 days, and ≥30 days of MV, respectively.

**Table 1:** The upper portion of the table outlines the demographic characteristics of participants. The lower portion of the table outlines the label distributions of participants, including patient count with and without each outcome before and after upsampling the low-frequency classes.


Characteristic		Cohort, No. (%)
Race	African American or Black	5,636 (54.31%)
	Asian	3,749 (36.12%)
	Caucasian or White	31 (0.30%)
	American Indian or Alaskan Native	23 (0.22%)
	Native Hawaiian or Other Pacific Islander	43 (0.41%)
	Multiple, Unknown, Unavailable, Unreported, Not Reported	896 (8.63%)
Ethnicity	Non-Hispanic or Latino	8,640 (83.25%)
	Hispanic or Latino	690 (6.65%)
	Unreported, Unknown, Unavailable, Not Recorded	1,048 (10.10%)
Gender	Female	5,281 (50.89%)
	Male	5,097 (49.11%)
BMI	<18.5	457 (4.40%)
	18.5–24.9	2065 (19.90%)
	24.9–29.9	2,904 (27.98%)
	>30	4,952 (47.72%)
Characteristic		Median [IQR]
Age		60 [48–72]

Class	Frequency, No. (%)					
	Pre-Upsampling				Post-Upsampling	
	Overall	Train	Validate	Test	Train	Validate
MV Duration						
0 days	8,141 (78.44)	4,907 (78.81)	1,609 (77.50)	1,625 (78.28)	4,907 (15.76)	1,609 (15.50)
1–4 days	812 (7.82)	469 (7.53)	175 (8.43)	168 (8.09)	3,916 (12.58)	1,583 (15.25)
5–9 days	325 (3.13)	200 (3.21)	60 (1.97)	65 (3.13)	3,313 (10.64)	1,098 (10.58)
10–14 days	241 (2.32)	165 (2.65)	41 (1.97)	35 (1.69)	3,899 (12.52)	1,079 (10.39)
15–19 days	153 (1.47)	84 (1.35)	38 (1.83)	31 (1.49)	3,502 (11.25)	1,438 (13.85)
20–24 days	97 (0.93)	56 (0.90)	20 (0.96)	21 (1.01)	3,445 (11.07)	1,058 (10.19)
25–29 days	87 (0.84)	50 (0.80)	17 (0.82)	20 (0.96)	3,163 (10.16)	1,055 (10.16)
30 + days	522 (5.03)	295 (4.74)	116 (5.59)	111 (5.35)	4,985 (16.01)	1,460 (14.07)
ECMO						
True	15 (0.14)	10 (0.16)	3 (0.14)	2 (0.10)	3,335 (10.71)	1,078 (10.39)
False	10,363 (99.86)	6,216 (99.84)	2073 (99.86)	2074 (99.90)	27,795 (89.29)	9,302 (89.61)
Mortality						
True	1,114 (10.73)	667 (10.71)	222 (10.69)	225 (10.84)	10,900 (35.01)	3,223 (31.05)
False	9,264 (89.27)	5,559 (89.29)	1854 (89.31)	1851 (89.16)	20,230 (64.99)	7,157 (68.95)

The lower portion of the table outlines the label distributions of participants, including patient count with and without each outcome before and after upsampling the low-frequency classes. Quality of evidence: Level 2.

Due to class imbalances, we perform random up-sampling for our training and validation datasets (Provost, 2000) of patients with low-frequency outcome classes (defined as outcome frequency < 15% with respect to the overall dataset), resulting in a bootstrapped dataset with outcome distributions listed in Table 1.

### 3.2 Model comparison

Table 2 depicts the results of the model performances on our holdout dataset, reporting AUROC, AUPRC, and F-score with respective confidence intervals (normal approximation intervals).

**Table 2:** This table depicts the MV duration, ECMO, and mortality prediction results for all three models.

Model performance						
Model	Class	F-score	PRAUC	AUROC	Precision	Recall
MV Duration						
RNN	Weighted Avg	0.688	**0.790**	**0.873**	0.785	0.626
	Macro Avg	0.229	0.214	**0.834**	0.221	0.266
	0 Days	0.823 (0.806–0.839)	0.968 (0.960–0.975)	0.891 (0.878–0.905)	0.958 (0.949–0.966)	0.721 (0.702–0.741)
	1–4 Days	0.238 (0.220–0.257)	0.164 (0.149–0.180)	0.742 (0.723–0.760)	0.171 (0.155–0.187)	0.393 (0.372–0.414)
	5–9 Days	0.118 (0.104–0.132)	0.091 (0.078–0.103)	0.765 (0.746–0.783)	0.087 (0.075–0.099)	0.185 (0.168–0.201)
	10–14 Days	0.104 (0.091–0.117)	0.063 (0.053–0.074)	0.837 (0.821–0.853)	0.075 (0.064–0.086)	0.171 (0.155–0.188)
	15–19 Days	0.103 (0.090–0.116)	0.078 (0.067–0.090)	0.890 (0.876–0.903)	0.076 (0.064–0.087)	0.161 (0.145–0.177)
	20–24 Days	0.102 (0.089–0.115)	0.045 (0.036–0.054)	0.784 (0.767–0.802)	0.079 (0.067–0.091)	0.143 (0.128–0.158)
	25–29 Days	0.045 (0.036–0.054)	0.048 (0.038–0.057)	0.881 (0.867–0.895)	0.042 (0.033–0.050)	0.050 (0.041–0.059)
	30 + Days	0.294 (0.275–0.314)	0.258 (0.239–0.277)	0.882 (0.868–0.896)	0.283 (0.264–0.303)	0.306 (0.286–0.326)
DT	Weighted Avg	**0.762**	0.775	0.812	**0.800**	**0.733**
	Macro Avg	**0.254**	0.184	0.613	**0.246**	**0.273**
	0 Days	0.904 (0.891–0.916)	0.953 (0.944–0.962)	0.865 (0.850–0.880)	0.962 (0.954–0.970)	0.852 (0.837–0.868)
	1–4 Days	0.336 (0.316–0.357)	0.194 (0.177–0.211)	0.672 (0.651–0.692)	0.268 (0.249–0.287)	0.452 (0.431–0.474)
	5–9 Days	0.100 (0.087–0.113)	0.042 (0.033–0.050)	0.537 (0.515–0.558)	0.093 (0.081–0.106)	0.108 (0.094–0.121)
	10–14 Days	0.083 (0.071–0.095)	0.026 (0.019–0.033)	0.552 (0.530–0.573)	0.059 (0.049–0.069)	0.143 (0.128–0.158)
	15–19 Days	0.032 (0.025–0.040)	0.016 (0.010–0.021)	0.509 (0.487–0.530)	0.032 (0.025–0.040)	0.032 (0.025–0.040)
	20–24 Days	0.051 (0.042–0.061)	0.012 (0.008–0.017)	0.520 (0.498–0.541)	0.056 (0.046–0.065)	0.048 (0.038–0.057)
	25–29 Days	0.143 (0.128–0.158)	0.025 (0.018–0.032)	0.570 (0.549–0.592)	0.136 (0.122–0.151)	0.150 (0.135–0.165)
	30 + Days	0.379 (0.358–0.400)	0.203 (0.186–0.220)	0.679 (0.659–0.699)	0.364 (0.343–0.384)	0.396 (0.375–0.417)
LR	Weighted Avg	0.651	0.780	0.727	0.769	0.577
	Macro Avg	0.202	**0.270**	0.592	0.196	0.251
	0 Days	0.787 (0.769–0.804)	0.959 (0.950–0.968)	0.769 (0.751–0.787)	0.947 (0.938–0.957)	0.673 (0.652–0.693)
	1–4 Days	0.211 (0.193–0.228)	0.150 (0.135–0.166)	0.578 (0.557–0.600)	0.171 (0.155–0.187)	0.274 (0.255–0.293)
	5–9 Days	0.139 (0.124–0.154)	0.105 (0.091–0.118)	0.590 (0.569–0.611)	0.094 (0.082–0.107)	0.262 (0.243–0.280)
	10–14 Days	0.116 (0.102–0.130)	0.087 (0.075–0.099)	0.601 (0.580–0.622)	0.075 (0.064–0.086)	0.257 (0.238–0.276)
	15–19 Days	0.032 (0.024–0.039)	0.034 (0.026–0.042)	0.510 (0.488–0.531)	0.021 (0.015–0.027)	0.065 (0.054–0.075)
	20–24 Days	0.031 (0.024–0.039)	0.051 (0.042–0.061)	0.514 (0.492–0.535)	0.023 (0.017–0.030)	0.048 (0.038–0.057)
	25–29 Days	0.118 (0.104–0.132)	0.063 (0.052–0.073)	0.589 (0.568–0.610)	0.083 (0.071–0.095)	0.200 (0.183–0.217)
	30 + Days	0.187 (0.170–0.204)	0.209 (0.192–0.227)	0.581 (0.560–0.602)	0.156 (0.140–0.171)	0.234 (0.216–0.252)
ECMO						
RNN	Weighted Avg	**0.997**	**0.999**	**0.902**	**0.998**	**0.997**
	Macro Avg	**0.499**	**0.504**	**0.902**	**0.500**	**0.499**
	True	0.000 (0.000–0.000)	0.007 (0.004–0.011)	0.902 (0.890–0.915)	0.000 (0.000–0.000)	0.000 (0.000–0.000)
	False	0.998 (0.997–1.000)	1.000 (0.999–1.000)	0.902 (0.890–0.915)	0.999 (0.998–1.000)	0.998 (0.995–1.000)
DT	Weighted Avg	**0.997**	0.998	0.498	**0.998**	0.996
	Macro Avg	**0.499**	0.500	0.498	**0.500**	0.498
	True	0.000 (0.000–0.000)	0.001 (0.000–0.002)	0.498 (0.477–0.520)	0.000 (0.000–0.000)	0.000 (0.000–0.000)
	False	0.998 (0.996–1.000)	0.999 (0.998–1.000)	0.498 (0.477–0.520)	0.999 (0.998–1.000)	0.997 (0.994–0.999)
LR	Weighted Avg	**0.997**	**0.999**	0.499	**0.998**	**0.997**
	Macro Avg	**0.499**	0.502	0.499	**0.500**	**0.499**
	True	0.000 (0.000–0.000)	0.005 (0.002–0.009)	0.499 (0.477–0.520)	0.000 (0.000–0.000)	0.000 (0.000–0.000)
	False	0.998 (0.997–1.000)	1.000 (0.999–1.000)	0.499 (0.477–0.520)	0.999 (0.998–1.000)	0.998 (0.995–1.000)
Mortality						
RNN	Weighted Avg	0.862	**0.893**	**0.774**	0.858	**0.866**
	Macro Avg	0.633	**0.637**	**0.774**	0.641	0.626
	True	0.340 (0.320–0.361)	0.312 (0.292–0.332)	0.774 (0.756–0.792)	0.364 (0.343–0.384)	0.320 (0.300–0.340)
	False	0.925 (0.914–0.936)	0.963 (0.955–0.971)	0.774 (0.756–0.792)	0.919 (0.907–0.930)	0.932 (0.921–0.943)
DT	Weighted Avg	**0.868**	0.860	0.669	**0.870**	**0.866**
	Macro Avg	**0.664**	0.600	0.669	**0.659**	**0.669**
	True	0.403 (0.382–0.425)	0.267 (0.248–0.286)	0.669 (0.649–0.689)	0.390 (0.369–0.411)	0.418 (0.397–0.439)
	False	0.925 (0.913–0.936)	0.993 (0.922–0.943)	0.669 (0.649–0.689)	0.929 (0.918–0.940)	0.921 (0.909–0.932)
LR	Weighted Avg	0.839	0.891	0.636	0.853	0.828
	Macro Avg	0.615	0.624	0.636	0.604	0.636
	True	0.330 (0.309–0.350)	0.284 (0.265–0.304)	0.636 (0.615–0.657)	0.285 (0.265–0.304)	0.391 (0.370–0.412)
	False	0.901 (0.888–0.914)	0.965 (0.957–0.973)	0.636 (0.615–0.657)	0.922 (0.911–0.934)	0.881 (0.867–0.895)

The highest weighted average and macro average results across each metric are bolded. Quality of evidence: Level 2.

AUROC: On mechanical ventilator duration, ECMO use, and mortality outcome, the RNN reached weighted average AUROC scores of 0.873, 0.902, and 0.774; the highest performing DT model reached weighted average AUROC scores of 0.812, 0.498, 0.669; and the highest performing LR model reached weighted average AUROC scores of 0.727, 0.499, and 0.636, respectively.

AUPRC: On mechanical ventilator duration, ECMO use, and mortality outcome, the RNN reached weighted average AUPRC scores of 0.790, 0.999, and 0.893; the highest performing DT model reached weighted average AUPRC scores of 0.775, 0.998, 0.860; and the highest performing LR model reached weighted average AUPRC scores of 0.780, 0.999, and 0.891, respectively.

F-score: On mechanical ventilator duration, ECMO use, and mortality outcome, the RNN reached weighted average F-score of 0.688, 0.997, and 0.862; the highest performing DT model reached weighted average AUROC scores of 0.762, 0.997, 0.868; and the highest performing LR model reached weighted average AUROC scores of 0.651, 0.997, and 0.839, respectively.

### 3.3 Feature importance

**Figure 3:**
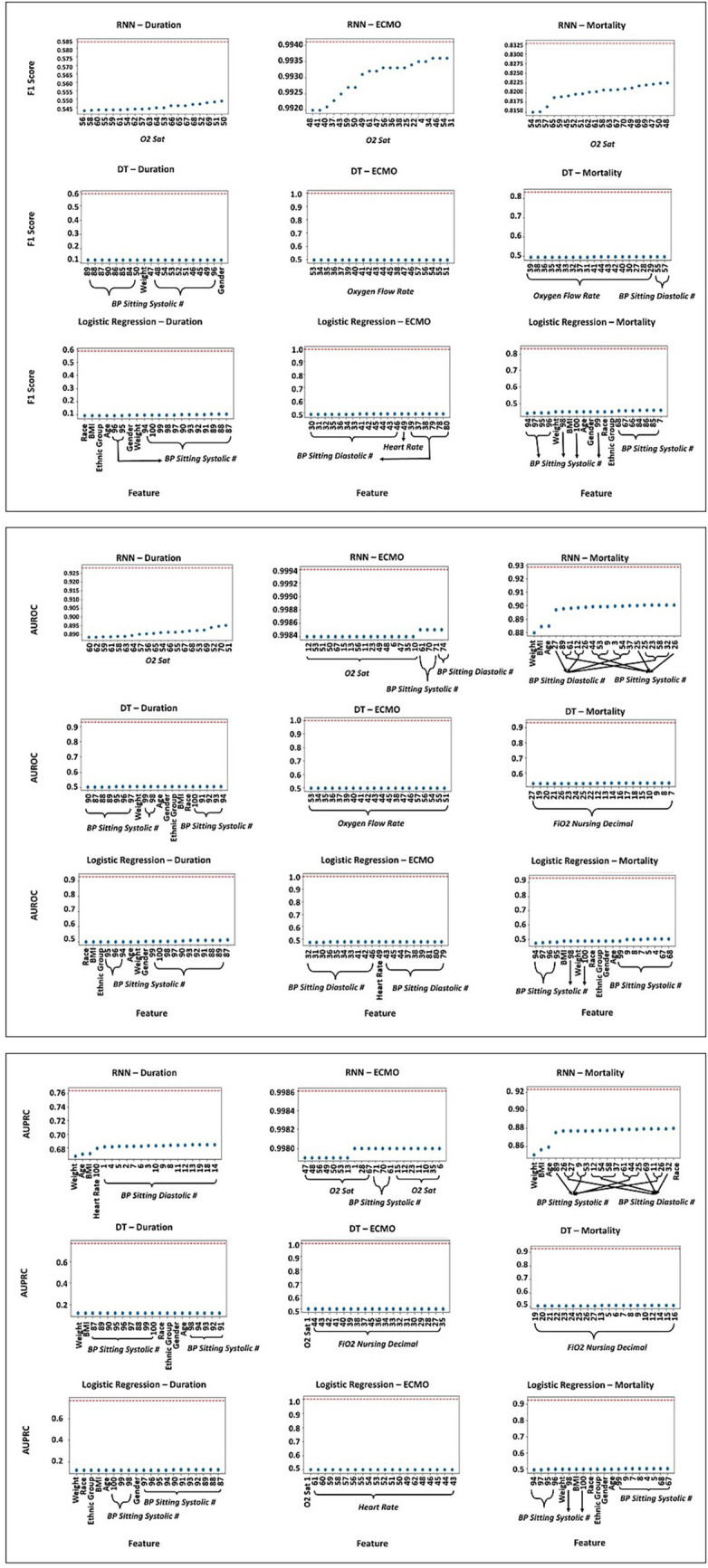
Feature permutation importance with respect to weighted average F-Score, AUROC, and AUPRC Score for RNN, DT, and LR models, for the MV duration, ECMO, and mortality outcomes. The *x*-axis lists feature names, and the *y*-axis captures model performance. Lower model performance indicates higher feature importance for that model. Nominal model performance (with no feature permutations) is indicated with the red dotted line. Note that while this figure reports individual metrics (F-Score, AUROC, AUPRC) across three different outcomes (ventilation duration, ECMO, and mortality), permuting a single feature may improve performance for one model’s outcome while degrading performance for another.

Following the PI procedure (Altmann et al., 2010), we randomly permute the feature column across patients of the holdout dataset 100 times, and evaluate the resulting model performance to evaluate feature importance (Figure 3). Each timestep of each dynamic feature is a distinct feature type. If the model performs poorly for a given permuted feature, this suggests that the feature is informative. See Supplementary material for SHAP analysis.

The RNN pipeline was most impacted by BP sitting systolic and diastolic measurements, heart rate, O_2_ saturation, age, BMI, race, and weight. As evaluated by F-score, the RNN pipeline was most impacted by O_2_ saturation. In addition to O_2_ saturation, the RNN pipeline, as evaluated by AUROC, was most impacted by BP sitting systolic and diastolic measurements, age, BMI, and weight. Heart rate and race were also important for the RNN pipeline as evaluated by AUPRC.

The DT and LR pipelines rely on a greater variety of features than the RNN. The DT pipeline was most impacted by BP sitting systolic and diastolic measurements, FiO_2_ nursing decimal, oxygen flow rate, O_2_ saturation, age, BMI, ethnic group, gender, race, and weight. From the feature permutation analysis, the DT pipeline, as evaluated by F-score, was most impacted by BP sitting systolic and diastolic measurements, oxygen flow rate, gender, and weight. The DT pipeline, as evaluated by AUROC, was most impacted by BP sitting systolic, FiO_2_ nursing decimal, oxygen flow rate, age, BMI, ethnic group, gender, race, and weight. The DT pipeline, as evaluated by AUPRC, was most impacted by most of the same features as AUROC, with the removal of oxygen flow rate and the addition of O_2_ saturation. From the SHAP analysis, we find that the DT pipeline was also strongly impacted by temperature and heart rate.

The LR pipeline was most impacted by O_2_ saturation, BP sitting systolic and diastolic measurements, heart rate, weight, race, age, BMI, ethnic group, and gender. From the feature permutation analysis, the LR pipeline, as evaluated by F-score and AUROC, was most impacted by BP sitting systolic and diastolic measurements, heart rate, age, BMI, ethnic group, gender, race, and weight. The LR pipeline, as evaluated by AUPRC, was most impacted by most of the same features, with the removal of BP sitting diastolic measurement and the addition of O_2_ saturation. From the SHAP analysis, we find that the LR pipeline was also strongly impacted by oxygen flow rate and FiO_2_ nursing decimal.

## 4 Discussion

In this retrospective, prognostic study we developed and validated three ML models on 10,378 COVID-19 patients to predict MV duration, as well as ECMO and mortality outcome, which is novel compared to other algorithms that did not examine all three as distinct outcomes in the same model.

### 4.1 Model performance in held-out cohort

The highest-performing model for the weighted average AUROC was the RNN, with MV duration, ECMO, and mortality AUROC of 0.873, 0.902, and 0.774. Similarly, the highest-performing model for macro average AUROC, which treats all classes as equally weighted when averaging, was the RNN, with MV duration, ECMO, and mortality AUROC of 0.834, 0.902, and 0.774, outperforming other models by a margin of 0.221 for MV duration, 0.403 for ECMO, and 0.105 for mortality.

The highest-performing model for the weighted average AUPRC was the RNN, with MV duration, ECMO, and mortality AUPRCs of 0.790, 0.999, and 0.893. For MV duration, the highest-performing model for macro average AUPRC was the LR with 0.270, outperforming the other models by a margin of 0.056. The highest-performing model for macro average AUPRC was the RNN for ECMO and mortality with AUROC of 0.504 and 0.637, outperforming other models by a margin of 0.002 for ECMO and 0.013 for mortality.

The highest-performing model with respect to weighted average F-score was the DT for MV duration and mortality, with F-scores of 0.762 and 0.868. The highest-performing model for macro average F-score was the DT for MV duration and mortality with F-scores of 0.254 and 0.664, outperforming the other models by a margin of 0.025 for MV duration and 0.031 for mortality. For ECMO, three models demonstrate equal performance with weighted average and macro average F-scores of 0.997 and 0.499.

Finally, the Brier scores for the RNN model’s MV duration, ECMO, and mortality predictions were 0.44, 0.01, and 0.22, respectively. The Brier scores for the DT model’s MV duration, ECMO, and mortality predictions were 0.47, 0.01, and 0.25, respectively. The Brier scores for the LR model’s MV duration, ECMO, and mortality predictions were 0.51, 0.01, and 0.24, respectively. While lower Brier scores indicate higher calibration, the low ECMO Brier scores observed are likely reflective of the class imbalance rather than superior model performance.

These findings suggest that the RNN architecture would be best suited for the task of predicting the duration of MV use, ECMO, and mortality in COVID-19 patients, as this model is the highest-performing with respect to weighted and macro averaged AUROC and weighted AUPRC. We hypothesize the RNN demonstrated higher performance as it was naturally able to incorporate time-series data and learn more complex representations of the data. In addition to the RNN’s ability to more effectively process temporal data, the RNN was the only model that backpropagated throughout the layers that predicted each outcome, performing end-to- end learning. This means that the different components of the model were learned together, rather than sequentially, resulting in higher performance. On the other hand, the LR and DT models were chained, training each component of the pipeline sequentially and independently. These factors may have contributed to the RNN’s superior model’s performance, compared to the DT and LR models.

### 4.2 Saliency analysis

We perform feature permutation among the models to identify factors important in all models. The strong predictors shared across all three models include BP sitting systolic and diastolic measurements, heart rate, O_2_ saturation, age, BMI, race, weight. The DT and LR pipelines rely on a greater variety of features than the RNN. This is notable, as a model that relies on fewer strong predictors requires fewer features to be collected to perform well, facilitating the data collection process. However, a model that relies on a larger variety of features may be more robust to missing data compared to a model that relies heavily on a constrained set of features. Additional strong predictors shared across only the DT and LR include FiO_2_ nursing decimal, oxygen flow rate, ethnic group, and gender.

### 4.3 Limitations and future work

We only kept individuals in our dataset that have at least one measurement for each data feature; however, this could be addressed with training procedures, e.g., random feature dropout. Further, our models do not perform real-time prediction; however, our RNN-based approach naturally affords the inclusion of additional data in real-time as it becomes available. For this paper, we focused on early detection, leveraging only the first three consecutive days of hospital visit data.

The highest-performing model in this work was the RNN. Transformer-based architectures, which also perform sequential processing, could be a promising option for future work. However, data-hungry Transformers require larger datasets, but transfer learning could help (Agarwal et al., 2022).

Additionally, while a patient may visit the hospital multiple times throughout their life, we do not incorporate previous visits medical data to improve our models’ predictions. Future work could investigate leveraging prior medical history to improve model accuracy.

Due to the scarcity of ECMO in our dataset, ECMO predictions may have limited clinical applicability. Furthermore, excluding patients without at least one measurement per feature type may bias our model toward more closely monitored or severe cases.

We include race and ethnic group as features in our model to mitigate bias, as our dataset’s demographics are not representative of the typical U. S. hospital population. Future work could evaluate model transferability to populations with different demographics and consider replacing race/ethnicity with structural and social determinants of health related to factors such as economic stability and access to healthcare and education.

Finally, future work could investigate real-time prediction and validation in a clinical deployment setting.

## 5 Conclusion

In conclusion, in this retrospective, prognostic study, we compare the performance of ML models trained on clinical variables and demographic information on the prediction of COVID- 19 outcomes. Our RNN-based approach was the highest- performing model for predicting mechanical ventilation duration (AUROC = 0.873, AUPRC = 0.790), extracorporeal membrane oxygenation (AUROC = 0.902, AUPRC = 0.999), and mortality (AUROC = 0.774, AUPRC = 0.893). This work suggests that hierarchical ML models have the potential to support clinicians in personalizing treatment and mitigating the risk of prolonged mechanical ventilation.

## Data Availability

The data analyzed in this study is subject to the following licenses/restrictions: qualified researchers may access data from the Emory Data Warehouse upon reasonable request and data use agreement. Requests to access these datasets should be directed to https://it.emory.edu/clinical-research-data/sources/warehouse.html.

## Supplementary material

SUPPLEMENTARY FIGURE 1 SHAP summary plots for the DT and LR models, for the MV duration, ECMO, and mortality outcomes.

SUPPLEMENTARY FIGURE 2 ROC plots for the RNN, DT, and LR models, for the mechanical ventilation duration, ECMO, and mortality outcomes. The closer the ROC curve is to the top left corner, the higher the model’s accuracy (higher true positive rate and lower false positive rate).

SUPPLEMENTARY FIGURE 3 Confusion matrices for the RNN, DT, and LR models, for the mechanical ventilation duration, ECMO, and mortality outcomes. The higher the diagonal values, the better the model performed.
