# Supplementary figures and images for: Use of machine learning models to predict mechanical ventilation, ECMO, and mortality in COVID-19
Source: Front Artif Intell. 2026 Jan 6;8:1661637. doi: 10.3389/frai.2025.1661637 (PMC12816323; doi:10.3389/frai.2025.1661637)

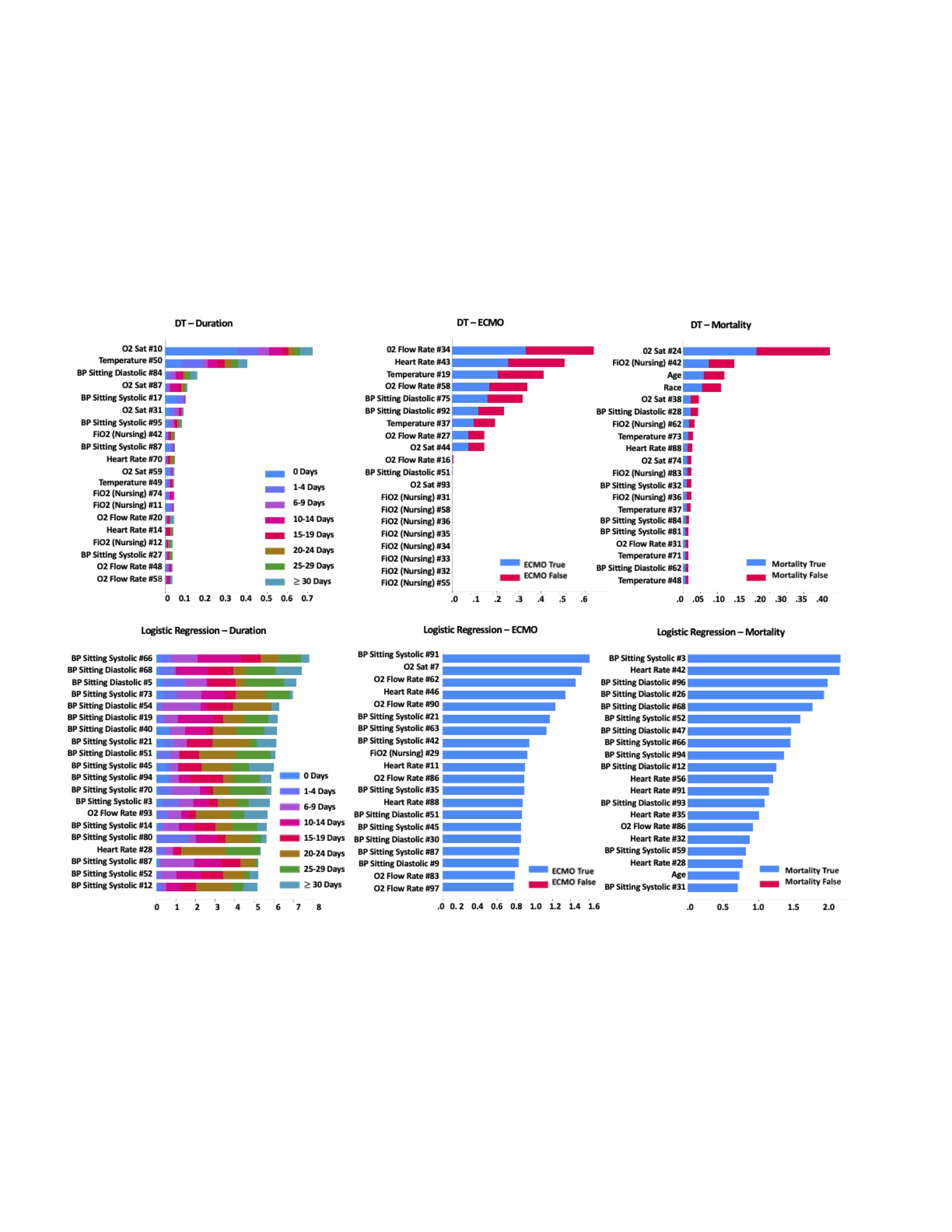

Supplement: SUPPLEMENTARY FIGURE 1 — SHAP summary plots for the DT and LR models, for the MV duration, ECMO, and mortality outcomes. [file Image_1.JPEG]

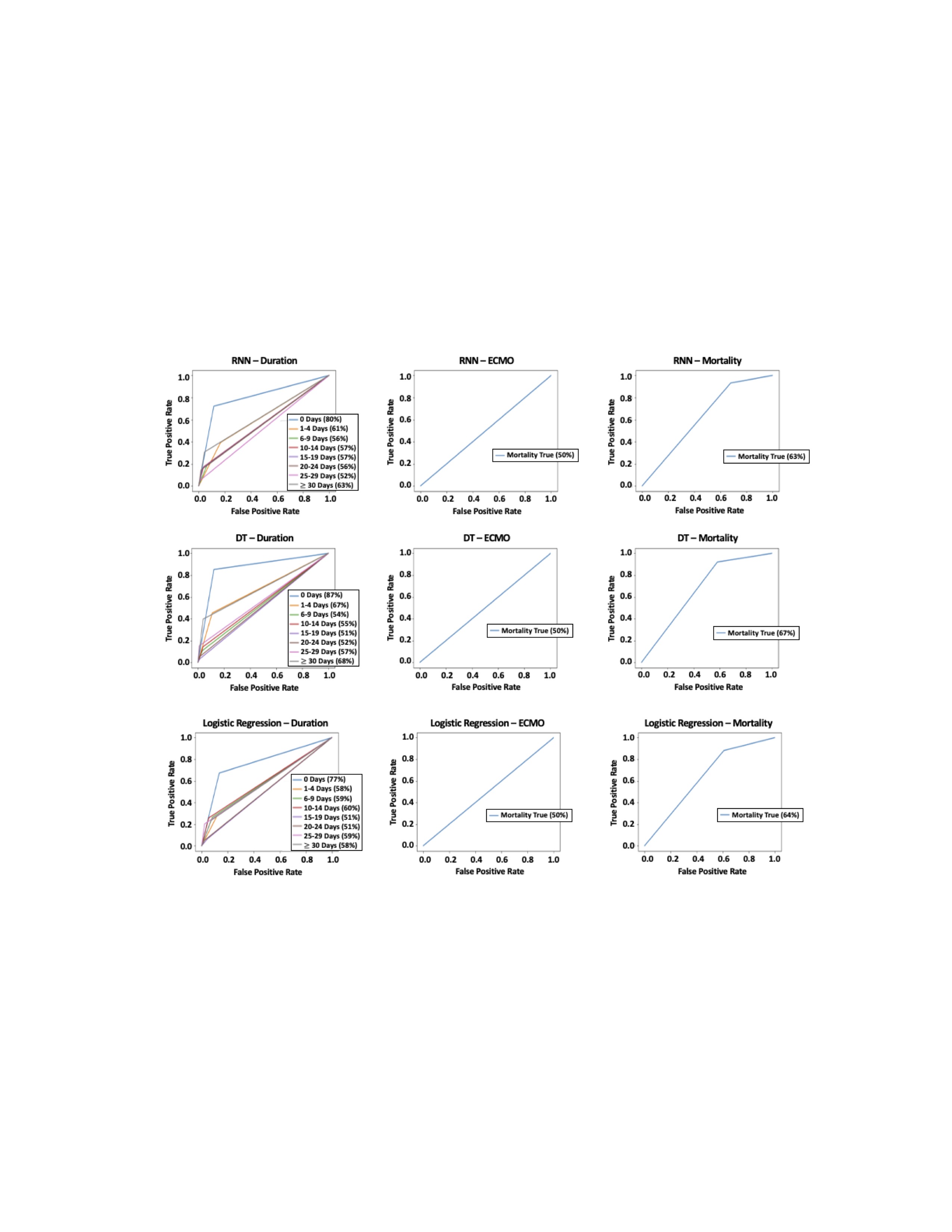

Supplement: SUPPLEMENTARY FIGURE 2 — ROC plots for the RNN, DT, and LR models, for the mechanical ventilation duration, ECMO, and mortality outcomes. The closer the ROC curve is to the top left corner, the higher the model’s accuracy (higher true positive rate and lower false positive rate). [file Image_2.JPEG]

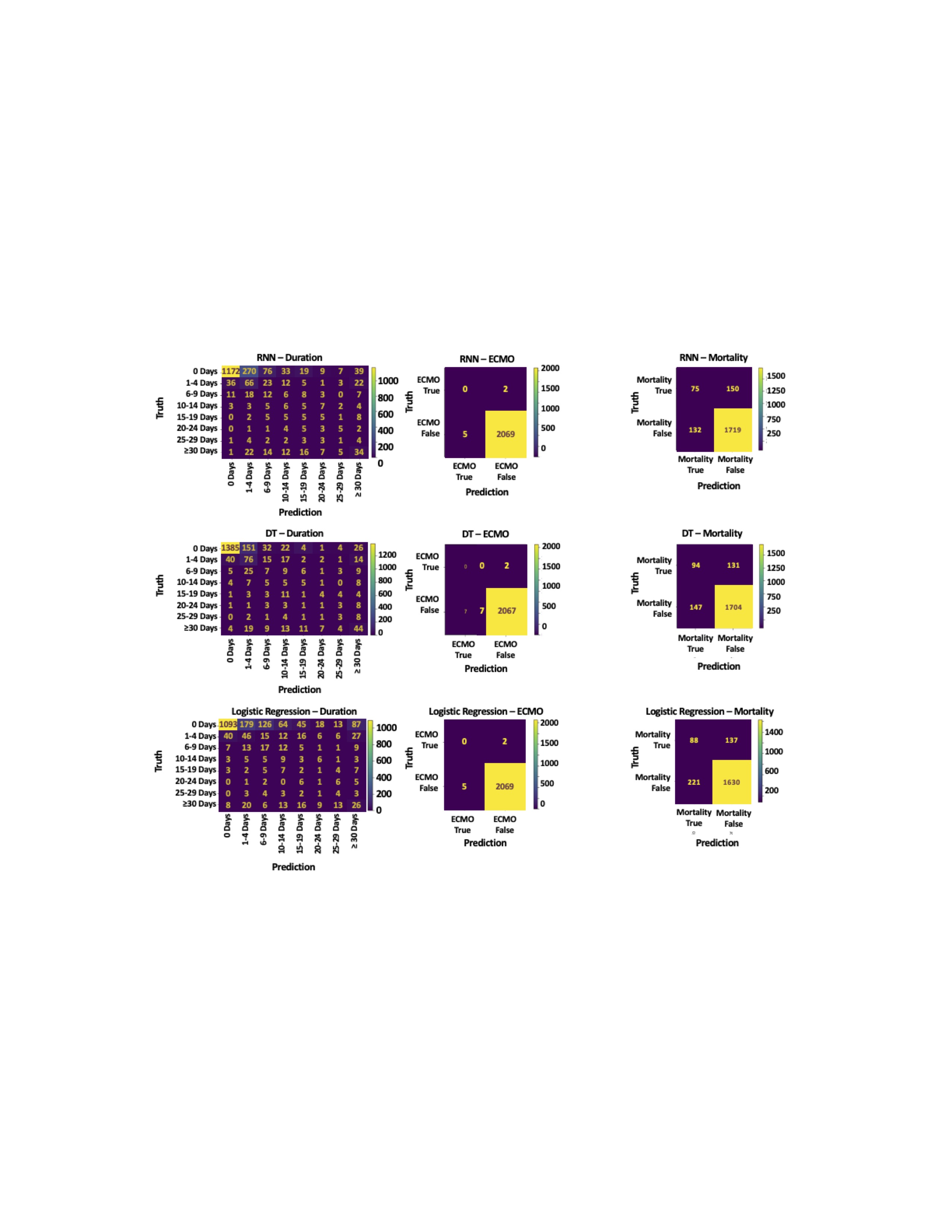

Supplement: SUPPLEMENTARY FIGURE 3 — Confusion matrices for the RNN, DT, and LR models, for the mechanical ventilation duration, ECMO, and mortality outcomes. The higher the diagonal values, the better the model performed. [file Image_3.JPEG]
