# Supplementary material for: Use of machine learning models to predict mechanical ventilation, ECMO, and mortality in COVID-19
Source: Front Artif Intell. 2026 Jan 6;8:1661637. doi: 10.3389/frai.2025.1661637 (PMC12816323; doi:10.3389/frai.2025.1661637)
Supplement: Supplementary file 4 [file Data_Sheet_1.docx]

**Supplementary**

## *Dataset*

*Features:* The categorical static feature categories are the following. For the race feature, the categories included African American or Black; Asian; Caucasian or White; American Indian or Alaskan Native; Native Hawaiian or Other Pacific Islander; Multiple; Unknown, Unavailable or Unreported; and Not Recorded. For the ethnic group feature, the categories included Non-Hispanic or Latino; Hispanic or Latino; Unreported, Unknown, Unavailable; and Not Recorded. For the gender feature, the categories included Female, Male, and Unknown. Finally, for the Body Mass Index feature, the categories included Under (*<* 18.5, Normal (*≥* 18.5 and *<* 25), Over (*≥* 25 and *<* 30), and Obese (*≥* 30).

*Labels:* The categorical duration outcome classes include 0 days, 1-4 days, 6-9 days, 10-14 days, 15-19 days, 20-24 days, 25-29 days, *≥*30 days. The 0 days bucket captures individuals who did not need mechanical ventilation, enabling us to predict both if mechanical ventilation will be needed, and if so, how long it will be needed. Both the ECMO and mortality outcome outputs have two classes: True and False, indicating whether the individual experienced this outcome during their hospitalization. We pre-process the ECMO and mortality labels to be one-hot encoded such that a positive ECMO or mortality label is [1, 0] and a negative ECMO or mortality label is [0, 1].

## *Model Development*

In this section, we provide further information regarding the grid search procedure.

**Decision Tree:** We perform a grid search over the following hyperparameters when fitting our decision tree model, with the class weight set to balanced, cross-validation set to 5- fold, and random state set to 0.

- max leaf nodes : [10, 25, 50, None]
- min samples leaf : [10, 25, 50, None]
- min samples split : [10, 25, 50, None]
- max depth : [10, 25, 50, None]
- criterion : [gini, entropy]

Decision trees are structured such that there is a root node, internal nodes that split into children, and leaf nodes that do not split further. The *max leaf nodes* parameter controls the complexity of the DT by limiting the total number of leaf nodes. The *min samples split* and *min samples leaf* parameters limit the complexity of the tree by controlling the minimum number of samples needed to justify a split, and the minimum number of samples needed for each resulting child of a split to justify a split. The *max depth* parameter controls the maximum depth (or level) of the tree, where shallow trees are less complex and deeper trees are more complex. Controlling the complexity of the DT through these parameters can help prevent overfitting. The last parameter, *criterion* is the criterion is used to measure the quality (such as impurity or error) of a split.

The resulting parameters for the decision tree pipeline were as follows.

- Mechanical ventilation duration decision tree model
- = *{class weight*: None, *criterion*: gini, *max depth*: 50, *max leaf nodes*: None, *min samples leaf* : 10, *min samples split*: 10, *random state*: 0*}*.
- ECMO decision tree model = *{class weight*: None, *criterion*: gini, *max depth*: 10, *max leaf nodes*: 25, *min samples leaf* : 25, *min samples split*: 10, *random state*: 0*}*.
- Mortality decision tree model = *{class weight*: None, *criterion*: entropy, *max depth*: 25, *max leaf nodes*: None, *min samples leaf* : 10, *min samples split*: 10, *random state*: 0*}*.

**Logistic Regression:** We perform a grid search over the following hyperparameters when fitting our logistic regression model, with the class weight set to balanced, penalty set to elastic net, solver set to sage, multi-class set to one-vs.-rest, max iterations set to 100, cv=5, and random state set to 0.

- C : [0.001, 0.01, 0.1]
- L1 ratio : [0.2, 0.5, 0.8]

The *C* parameter controls the amount of regularisation in the model, with its inverse 1/C denoted as regularization strength. The *L1 ratio* parameter ranges from 0 to 1 and weighs the l1 vs l2 regularisation for elasticnet regularization.

The resulting parameters for the logistic regression pipeline were as follows.

- Mechanical ventilation duration logistic regression model = *{C*: 0.1, *class weight*: None, *l1 ratio*: 0.5, *max iter*: 100, *multi class*: ovr, *penalty*: elasticnet, *random state*: 0, *solver*: saga*}*.
- ECMO logistic regression model = *{C*: 0.01, *class weight*: None, *l1 ratio*: 0.2, *max iter*: 100, *multi class*: ovr, *penalty*: elasticnet, *random state*: 0, *solver*: saga*}*.
- Mortality logistic regression model = *{C*: 0.1, *class weight*: None, *l1 ratio*: 0.2, *max iter*: 100, *multi class*: ovr, *penalty*: elasticnet, *random state*: 0, *solver*: saga*}*.

**RNN:** The final RNN model hyperparameters employed are *{epochs*: 50, *batch size*: 64, *learning rate*: 1e-5, *dropout rate*: 0.2*}*.

## *Result Visualizations*

We now provide additional figures to visualize our results.

*SHapley Additive exPlanations* SHapley Additive exPlanations (SHAP) is a model-agnostic method for estimating the effect that each feature has on the outcome prediction(51, 52). The metric that captures how much the feature contributed to a prediction is termed the SHAP value. SHAP values are obtained by averaging, over all possible scenarios (i.e. all possible feature combinations), how much a feature changes the prediction of the model. Using the shap Python API, for each model we obtain summary plots of the SHAP values for the MV duration, ECMO, and mortality outcomes, as depicted in Supplemental Figure 1.

**SHAP Results for the DT Pipeline:** With respect to dynamic features, O2 saturation, oxygen flow rate, BP sitting systolic and diastolic, heart rate, and temperature are the strong predictors (SHAP value above 0.2) for the DT pipeline. Breaking down the component-level feature importance, we find that:

- O2 saturation and temperature served as strong predictors for the MV duration DT model. Other feature types in the top 20 most important features of the MV duration DT model include blood pressure (BP) sitting systolic and BP sitting diastolic, FiO2 nursing decimal, heart rate, and oxygen flow rate.
- Oxygen flow rate, heart rate, temperature, oxygen flow rate, and blood pressure sitting diastolic served as strong predictors to the ECMO DT model. Other feature types in the top 20 most important features of the ECMO DT model include O2 saturation and FiO2 nursing decimal.
- The mortality DT model relies strongly on O2 saturation to make predictions. Other feature types in the top 20 most important features of the mortality DT model include FiO2 nursing decimal, age, race, heart rate, temperature, and BP sitting diastolic/ systolic.

**SHAP Results for the LR Pipeline:** With respect to dynamic features, BP sitting systolic and diastolic, heart rate, O2 saturation, FiO2 nursing decimal, and oxygen flow rate are strong predictors for the LR pipeline. With respect to static features, age is the only strong predictor for the mortality LR component of the LR pipeline (as is observed in the DT pipeline). Breaking down the component-level feature importance, we find that:

- BP sitting systolic, BP sitting diastolic, oxygen flow rate, and heart rate are strong predictors for the MV duration LR model.
- BP sitting systolic, O2 saturation, oxygen flow rate, heart rate, BP sitting diastolic, and FiO2 nursing decimal are strong predictors for the ECMO LR model.
- BP sitting systolic, heart rate, BP sitting diastolic, oxygen flow rate, and age are strong predictors for the mortality LR model.

**SHAP Results for the RNN Pipeline:** Due to the inclusion of LSTM layers in the RNN architecture, and due to the design of our architecture which integrates static and dynamic inputs at different points in the network, rather than concatenating them at the first layer, our architecture is not supported by the shap Python API. As such, we do not report SHAP results for our RNN model. To enable comparisons of feature importance between our RNN model, DT, and LR models, we further provide permutation importance results.

*Receiver Operating Characteristic Plots* The receiver operating characteristic (ROC) Plots in Supplemental Figure 2, visualize how well each model can discriminate between the classes of each outcome. The AUROC is listed for each class, for each plot. For the mechanical ventilation outcome, we evaluate how well the model can tell apart each class, from all the other classes combined (one-vs-rest). As the ECMO and mortality outcomes are either true or false, we simply evaluate how the model can tell apart true outcomes from false outcomes.

*Confusion Matricies* Finally, the confusion matrices in Supplemental Figure [3](#_bookmark63) provide a summary of the prediction results, depicting the rate of true positive, true negative, false positive, and false negative predictions of each model for each class of each outcome.

**Supplementary Figure 1.** SHAP summary plots for the DT and LR models, for the MV duration, ECMO, and mortality outcomes.

**Supplementary Figure 2.** ROC plots for the RNN, DT, and LR models, for the mechanical ventilation duration, ECMO, and mortality outcomes. The closer the ROC curve is to the top left corner, the higher the model’s accuracy (higher true positive rate and lower false positive rate).

**Supplementary Figure 3.** Confusion matrices for the RNN, DT, and LR models, for the mechanical ventilation duration, ECMO, and mortality outcomes. The higher the diagonal values, the better the model performed.
